# *Leishmania major* virulence attenuation *in vitro*: An old conundrum revisited in the omics era

**DOI:** 10.1371/journal.pntd.0014387

**Published:** 2026-05-29

**Authors:** Evgeny S. Gerasimov, Kristína Záhonová, Aygul Ishemgulova, Tatiana S. Novozhilova, Sai Kumar Mishra, Natalya Kraeva, Jovana Sádlová, Petr Volf, Sara L. Zimmer, Vyacheslav Yurchenko

**Affiliations:** 1 Department of Molecular Biology, Faculty of Biology, M.V. Lomonosov Moscow State University, Moscow, Russia; 2 Life Science Research Centre, Faculty of Science, University of Ostrava, Ostrava, Czechia; 3 Institute of Parasitology, Biology Centre, Czech Academy of Sciences, České Budějovice, Czechia; 4 Department of Parasitology, Faculty of Science, Charles University, BIOCEV, Vestec, Czechia; 5 Division of Infectious Diseases, Faculty of Medicine and Dentistry, University of Alberta, Edmonton, Canada; 6 Department of Parasitology, Faculty of Science, Charles University, Prague, Czechia; 7 Medical School Duluth Campus, University of Minnesota, Duluth, Minnesota, United States of America; Academic Medical Center: Amsterdam UMC Locatie AMC, NETHERLANDS, KINGDOM OF THE

## Abstract

**Background:**

It has been known for decades that long-term cultivation of *Leishmania in vitro* frequently leads to a loss of virulence, which is attributed to the selective advantage of avirulent subpopulations that outgrow the virulent ones. In the case of *L. major*, avirulent parasites retained the ability, albeit reduced, to develop nearly normally in sand fly vectors; however, they could not induce lesions in BALB/c mice. Residual persistence in the inguinal lymph nodes permitted re-isolation and subsequent additional murine passages of these flagellates. While a parasite line obtained after five consecutive passages of avirulent *Leishmania* in mice was not fully restored to virulence, this line, as well avirulent parasites passaged five times through the sand fly vector *Phlebotomus duboscqi* developed very efficiently in sand flies.

**Materials and methods:**

*Leishmania major* cell lines with differing capacities for host survival were studied using genomic and transcriptomic approaches. Specifically, we focused on genetic mutations, gene copy number variation, differential gene expression, and differences in kinetoplast DNA, including RNA editing and minicircle repertoire.

**Results:**

While genetic mutations contributed little to differences in host survival, changes in the gene copy number were correlated with alterations to avirulence. Survival capacity strongly correlated with gene expression patterns. Avirulent parasites showed increased abundance of ribosomal and translation-related transcripts compared with lines capable of persistent survival, suggesting selective pressure to restrict translational capacity in hosts. Most interestingly, the relative abundance of kinetoplast DNA minicircle classes, encoding guide RNAs, was altered during culture but reverted to the virulent pattern following mouse and sand fly passage.

**Conclusions:**

Our results indicate that at the DNA and mRNA levels, *L. major* survival in the insect vector or mammalian host is primarily driven by adaptive regulation of gene expression rather than fixed genetic changes. Modulation of translational capacity and host-specific expression programs appear central to parasite persistence, highlighting flexible cellular strategies that support survival in natural transmission cycles.

## Author summary

The unicellular parasite *Leishmania major* is transmitted by sand flies and typically causes cutaneous lesions in mammalian species, including humans and mice. Identification of the virulence factors that allow it to survive and replicate in the host environments is important for understanding cutaneous leishmaniasis and for the development of new prevention and treatment strategies. *Leishmania major* can grow in a liquid culture in the laboratory. The parasite growing long-term in this environment can acquire a reduced ability to replicate in sand flies and cause lesions in mammalian hosts. However, elements of virulence can be partially rescued in culture-grown *L. major* by subsequent passages in either of its hosts. Comparison of the original isolate, the long-term culture-grown strain, and its subsequently host-passaged descendent strains can reveal parasite gene regulation relevant to virulence. In this work, we perform a global gene expression analysis of these strains and identify gene products and protein categories with expression patterns that suggest that they may be *Leishmania* virulence factors.

## Introduction

It has been known for decades that various strains of the same *Leishmania* species, and even clones of a single strain, often differ in their ability to infect a host [1–4]. Long-term cultivation of these parasites *in vitro* frequently leads to a loss of virulence, which is attributed to the selective advantage of avirulent subpopulations that outgrow the virulent ones [5–8]. This attenuation of virulence may be (at least, partially) reversible, as passage through a susceptible host can lead to increased virulence in some species [9]. Modern genomic techniques allow us an inexpensive approach to uncover potential mechanisms facilitating the loss of virulence in attenuated *Leishmania* strains [10,11] that can be subsequently pursued in greater detail.

*Leishmania major* strain LV561 (MHOM/IL/67/LRC-L137 Jericho-II) was originally isolated from a cutaneous leishmaniasis (CL) patient in Israel; it induces rapidly growing lesions in infected mice [12–14]. An avirulent line (AV) was derived from LV561 through long-term *in vitro* cultivation. It retained the ability to develop nearly normally in sand fly vectors [13,15]. However, this line could not induce lesions in BALB/c mice, although parasite persistence in the inguinal lymph nodes permitted its re-isolation and subsequent additional murine passages. While a parasite line obtained after five consecutive passages of AV in mice (AVM) was not fully restored to virulence, the proportion of successful positive post-mortem cultures increased from 33% to 67%. In parallel, passage of AV five times through the sand fly vector *Phlebotomus duboscqi* yielded a cell line (AVS) that developed more efficiently in sand flies than both the AV and AVM lines [13].

Phenotypically, the AV line exhibited reduced expression of metacyclic lipophosphoglycan (LPG), and decreased expression and activity of the surface metalloprotease GP63 [16]. LPG and GP63 are GPI-anchored glycoconjugates, key components of the parasite’s surface glycocalyx that are critical for survival in vertebrate hosts [17,18]. In sand fly vectors, the role of *L. major* LPG is well established [19–22], while the role of GP63 remains less clear [23–25]. GP63 proteolytic activity was restored in both AVM and AVS lines, while LPG levels remained low [13,16]. Thus, it seems likely that some, but not all, protein expression and enzyme activity modifications resulting from culture growth are at least partially restored upon serial passage in either sand fly or murine host.

The primary goal of the current study was to compare the genomic sequences and mRNA expression of these partially characterized LV561-derived *L. major* lines to identify additional genes and pathways potentially involved in alterations in host persistence and virulence. We hypothesized that at least some of the changes observed in the AV line relative to the original virulent LV561 would prove to be reversible by serial passage in sand fly or mouse. The methods used here are insufficient to capture changes to virulence resulting from later steps in expression control, such as alterations to specific mRNA translation rates, or protein stability or modification. However, genetic changes, if identified, can indicate pathways or sets of proteins particularly relevant to virulence. We found that while mitochondrial editing appears largely unaffected across the cell lines, unexpectedly, passage through mice or sand fly results in a resetting of AV relative mitochondrial minicircle class abundances to be much more consistent with that of V line parasites. In addition, we observed the differential gene expression pattern in AVM relative to the AV line strongly mimicked the expression differences between V and AV lines. This demonstrates that mRNA-level expression differences were highly reversible. The up-regulated genes in V and AVM lines spanned multiple processes, including those related to the expression of surface molecules. Proteomic analyses may have some advantages to identify components of virulence. Nevertheless, our analyses at the genomic and transcriptomic level revealed pathways that in the future should be functionally validated as important for *L. major* survival in its hosts.

## Methods

### Parasites and cultivation

Four different lines of the *Leishmania major* strain LV561 (MHOM/IL/67/LRC-L137 Jericho-II) were investigated: the virulent line (V) freshly re-isolated from BALB/c mice, the avirulent line (AV) attenuated by 101 *in vitro* passages in culture, and the lines AVM and AVS that were obtained from the AV line by five subsequently passages in mice (AVM) and sand flies *Phlebotomus duboscqi* (AVS) [13]. All the lines were tested previously for virulence in BALB/c mice and development in sand flies. Prior to the molecular analysis described here, the lines were maintained in a cryo-bank at Charles University, Prague: AV frozen as p101 in 2014, V frozen as p1 (1 passage *in vitro* after isolation from mice) in 1996, AVM frozen as p3 (3 passages *in vitro* after 5 passages in mice) in 1999, and AVS frozen as p5 (5 passages *in vitro* after 5 passages in sand flies) in 1999. Additional *in vitro* passaging was avoided. Lines V and AV were retested in 2018 and 2020 and their phenotypes were confirmed. After thawing, parasites were passaged once in M199 medium (Sigma-Aldrich/ Merck, St. Louis, USA) supplemented with 10% heat-inactivated fetal calf serum (Thermo Fisher Scientific, Waltham, USA), 2% sterile urine, 1% Basal Medium Eagle vitamins (MP Biomedicals, Irvine, USA), and 250 μg/ml amikacin (Medochemie, Prague, Czechia) as described previously [26] and analyzed. The identity of species was validated as in [27].

### Nucleic acid isolation, library preparation and sequencing

Nucleic acids (DNA and RNA) were isolated as described previously [28] and sequenced at Macrogen Europe (Amsterdam, the Netherlands). The TruSeq DNA PCR-free protocol was used for the genomic DNA library that was sequenced on a NovaSeq 6000 resulting in ~55M 150 bp paired-end reads. Three TruSeq stranded mRNA libraries per line were sequenced on the same platform resulting in ~125M 150 bp paired-end reads each. Raw sequencing reads were deposited in the NCBI database under BioProject accession PRJNA1391897.

### Read processing

Sequencing reads were quality-checked with FastQC v. 0.12.0 [29] and trimmed with fastp v. 0.25.0 [30]. Specific flags ‘--trim_poly_g’, ‘--trim_poly_x’, ‘--cut-front’, ‘--cut_tail’, ‘--cut_window_size 4’ and ‘--cut_mean_quality 20’ were used to properly handle 2-dye chemistry sequencing output, ‘--length_required 100’ was employed to preserve information for unique read position assignment and get higher contiguity of *de novo* assemblies. The same settings were applied to transcriptomic reads, except for the ‘cut_front_mean_quality’, ‘cut_tail_mean_quality’, and ‘average_qual’ set to defaults, ‘length_required’ set to 50.

### De novo nuclear genome assembly and variant detection

Sample data were assembled *de novo* using SPAdes v. 4.2.0 [31]. Assembly completeness was assessed with BUSCO v. 6.0.0 [32]. Sequencing reads were mapped back on each genome’s assembly with Burrows-Wheeler Aligner (BWA) v. 0.7.19 [33] using ‘MEM’ algorithm with default settings. BAM files were processed with SAMtools v. 1.22 [34], alignment files were visualized in IGV v. 2.16.0 [35] to perform SNP quality curation. Variant calling was performed by a pileup method in BCFtools v. 1.22 [34] and FreeBayes v. 1.13.10 [36], both with default settings. BCFtools determined SNPs with ‘QUAL <= 20’ were filtered out and only the intersection of homozygous SNP sets from BCFtools and FreeBayes was further used. Correlations between samples were analyzed and plotted using a custom R script and ggplot2 R library v. 4.0.1 [37] for visualization.

### Reference-based variant detection and copy-number variation analysis of the nuclear genome

Trimmed and quality-checked sequencing reads were mapped on the reference genome of *Leishmania major* Friedlin from the TriTrypDB release 58 [38] with the BWA-MEM algorithm. Alignments were processed with SAMtools and variant calling was performed with FreeBayes. Variant filtering and inter-sample comparisons were done with BCFtools ‘view’ and ‘isec’ commands and the output was processed with custom python scripts into the final tables. Only homozygous (or nearly homozygous with allele frequency over 0.8) SNPs were analyzed. BEDtools v. 2.31.1 [39] were used for various genomic region manipulations and SNP counting. Gene coding regions along with 1,000 nt upstream and downstream flanking sequences were considered when assigning the SNPs to genes. Regions of interest were extracted and inspected in IGV to confirm the presence of SNPs.

Large structural variants were detected with the DELLY v. 1.5.0 [40] ‘call’ algorithm using the same bam mappings and visualized using a custom R script and gridExtra R library v. 2.3 [41]. All samples were collectively used to detect locus copy number variation with cn.MOPS R package [42] with window length values 300; 1,000; 5,000; and 10,000, and the ‘DNAcopy’ algorithm using the same bam mappings.

### Comparison of mitochondrial genomes and their expression

The BLASTN program of NCBI-BLAST suite v. 2.15.0+ [43] was used to search for the known maxicircle coding region (CR) sequence of *Leishmania* spp. in *de novo* assembled contigs of the V line. The coding region was extracted, re-oriented to start with the 1*2S rRNA* gene on the forward strand, and annotated by homology with *Leishmania* spp. The RNA sequencing reads were mapped and processed with T-Aligner and T-Aligner DE pipelines, as described previously [44,45]. Briefly, reads were mapped onto each gene or cryptogene sequence possessing short flanking sequences of ~50 bp. Predicted RNA editing and expression profiles and edited mRNA sequences were obtained with ‘findorfs’ and ‘coverage’ tools of the T-Aligner suite. The differential expression analyses of mitochondrial transcripts were performed on each pair of samples (in triplicates) using EdgeR [46] to detect differentially edited states with adjusted *p*-value significance threshold 0.001 and editing site support absolute fold change > 2. Visualization was done with T-Aligner scripts for differential editing analyses.

### Differential expression analysis of the nuclear genome

RNA sequencing reads were quality-checked with FastQC and MultiQC v. 1.31 [47] and trimmed with fastp. Trimmed reads were mapped onto the reference genome of *L. major* Friedlin from TriTrypDB with STAR v. 2.7.11b [48]. Produced and sorted bam files were quality-checked with Qualimap v. 2.3 [49]. Read counts were obtained with featureCounts v. 2.0.6 [50]. Differential expression was analyzed with DESeq2 package [51], using the simple ‘control versus experiment’ setup, where sample groups were provided in three biological replicates (3 versus 3). Genes with a fold change of ≥ 2 and an adjusted *p*-value of ≤ 0.001 were considered differentially expressed. Protein products conceptually translated from the differently expressed genes of all lineages were submitted to STRING v. 12.0 [52] and the network reconstruction was performed as described previously [53]. The same set of proteins was submitted to the eggNOG-mapper v. 2.1.12 [54] to identify potential functions of proteins using the clusters of orthologous genes (COG) categories. In total, 195, 185, and 129 differentially expressed genes were identified in the V vs AV, AVM vs AV, and AVS vs AV comparisons, respectively, of which 152, 144, and 98 genes carried a COG annotation. Differentially expressed genes lacking annotation or assigned to the “function unknown” category (S) constituted ~30–40% of the total across all comparisons, while “general function” category (R) was not part of the reference genome annotation table. Neither group was analyzed further. To assess whether differentially expressed genes were non-randomly distributed across functional categories, we performed one-sided Fisher’s exact tests for enrichment against the 4,754 COG-annotated genes of the *L. major* Friedlin reference genome, with Benjamini–Hochberg false discovery rate (FDR) correction applied within each comparison (S1 Table). The RRMScorer [55] was used to analyze the probability of RNA binding and preferences of differentially expressed proteins LmjF.18.0170 and LmjF.18.0190, which were also analyzed for nucleic acid and amino acid similarity and identity using the Needleman-Wunsch algorithm through EMBL-EBI [56]. MEME tool from the MEME suite [57] was used to detect potential motifs of sizes from 5 bp to 12 bp for the 217 differentially expressed genes of the V and AVM lines.

## Results

### Changes in the gene copy number are likely involved in alterations to avirulence

Single nucleotide polymorphisms (SNPs) are an obvious and testable source of phenotypic variation. Therefore, we compared genomic sequences between parasite lines to find homozygous SNPs, using the *L. major* Friedlin high-quality reference genome [58] for calling. In parallel, SNPs were identified using a *de novo* assembled V line, as the sequence divergence between Friedlin and LV561 strains could potentially cause mapping artifacts. Hundreds of SNPs between genomes of the LV561-derived lines and *L. major* Friedlin reference were detected. However, patterns of the variant calls among LV561-derived lines suggested that most were artifacts: most SNPs were found in tandemly repeated or long genes composed of internal repeated sequences, such as proteophosphoglycans. This genomic context is known to trigger technical artifacts during the alignment process [59–61]. Coverage variation results in SNPs called because of reads mapping to multiple locations, for which allele frequency floats across samples. These may be computationally identified as homozygous, heterozygous, or even absent SNPs across samples. Particularly when coverage is low, this can result in the identification of homozygous SNP false positives. Following manual curation to remove these, no cell line-specific homozygous SNPs were identified. Homozygous SNPs of all LV561-derived lines relative to the reference genome were identical, so we concluded that *de novo* mutations are not responsible for virulence attenuation.

Next, we analyzed parasite line-specific locus copy number variation (CNV) [16,62–65]. Only five loci exhibited statistically significant CNV among parasite lines (S1 Table). A chromosome 17 region is absent in the AVS line, eliminating several copies of a gene encoding the eukaryotic elongation factor 1-alpha (eEF1A). Also notable is a 7 kb locus of chromosome 34 with a copy number reduction in the AV relative to the V, AVM, and AVS lines. It contains a single gene (*LmjF.34.2380*) of unknown function. The protein encoded by *LmjF.34.2380* appeared in Trypanosomatidae after the separation of *Trypanosoma* lineage with several independent losses. It does not contain any predicted domain and it is likely cytosolic (S1 Table, tab LmjF.34.2380).

The largest and, arguably, the most interesting CNV region is overrepresented in the AV line. This 12 kb-long locus on chromosome 35 contains genes encoding 60S ribosomal protein L37 (LmjF.35.5100), RNA pseudouridylate synthase (LmjF.35.5110), biopterin transporter 1 (LmjF.35.5140 and LmjF.35.5150), tRNA (guanine-N(7)-)-methyltransferase (LmjF.35.5230), alpha-1,2-mannosyltransferase (LmjF.35.5250), isopentenyl-diphosphate delta-isomerase (LmjF.35.5330), amino acid permease (LmjF.35.5350 and LmjF.35.5360), as well as several genes encoding proteins of unknown function. Many of these proteins have been implicated in *Leishmania* spp. virulence [66,67]. Of special interest are the isopentenyl-diphosphate delta-isomerase shown in a GP63^high^ fraction of *L. amazonensis* exosomes to be implicated in enhanced cutaneous leishmaniasis development [68], and biopterin transporter 1. The latter was previously identified to be a part of the *LD1* (*Leishmania* DNA 1) locus [69–71] and its overexpression has been linked to adaptations to *in vitro* cultivation conditions and loss of virulence in different *Leishmania* spp. [72–75], similar to the AV line. This increase in copy number is correlated with the upregulation of transcripts of nearly all these genes two to four-fold in the AV line relative to the V line (S2 Table). Thus, it is likely that the AV line’s CNV is the mechanism, through which this gene dosage change is affected. However, additional experiments are necessary to determine whether the chromosome 35 CNV results in a changed growth or protein translation rate that impacts the parasite’s ability to grow in host organisms.

In summary, while SNPs in the nuclear genome appear unlikely to drive alterations in parasite virulence in different hosts, some CNV-facilitated gene dosage differences are likely involved in this process.

### Passage of the avirulent strain in mice restores gene expression nearly to that of the original virulent strain

Next, we analyzed whether the differential gene expression could account for the changes in virulence or persistence phenotypes across different parasite cell lines. As regulation at the transcriptional level is largely absent in trypanosomatids [76,77], differences in mRNA expression between cell lines likely result from altered mRNA stability. To analyze this, we performed high-throughput RNA sequencing followed by pairwise analyses of mRNA abundance in V, AVS, and AVM lines relative to the AV line (S2 Table). In the V line, 192 transcripts of protein coding loci were at least two-fold differentially expressed in comparison to the AV line (S2A Table). Nearly this many (182) were differentially expressed in the AVM line (S2B Table), whereas only 127 were differentially expressed in the AVS line compared to the AV counterpart (S2C Table). Twenty-three percent of all differentially expressed transcripts (71 out of 311) were annotated as coding for “hypothetical protein” and not studied further.

**Fig 1:**
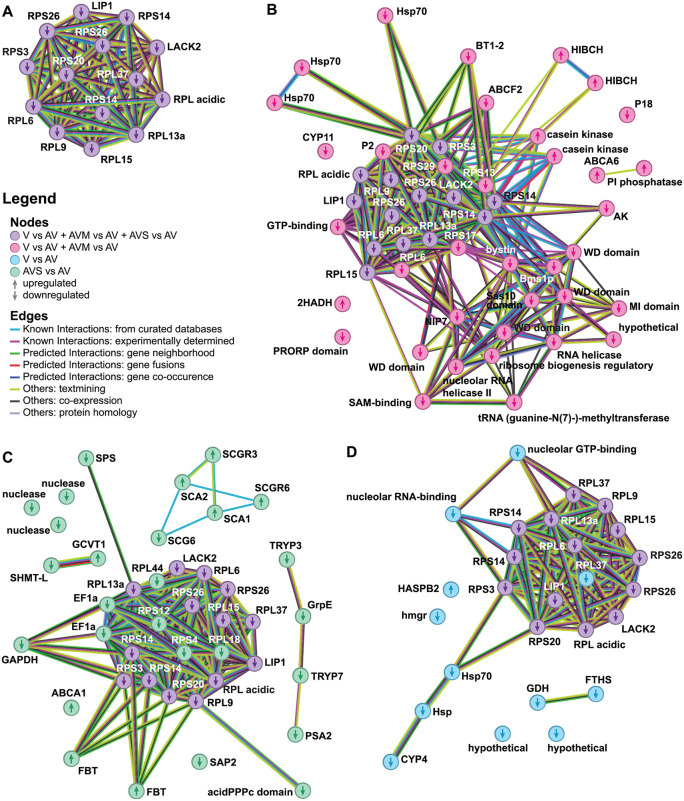
Protein comparison of differently expressed genes in studied cell lines. The protein network as generated by STRING shows changes in mRNA abundance between **(A)** V, AVM, and AVS versus AV; **(B)** V and AVM versus AV; **(C)** AVS versus AV; and **(D)** V versus AV. Nodes and edges represent *L. major* proteins and known or predicted interactions among them. Upward and downward arrows indicate up- or downregulation of the gene. For the full network see S1 Fig.

**Fig 2:**
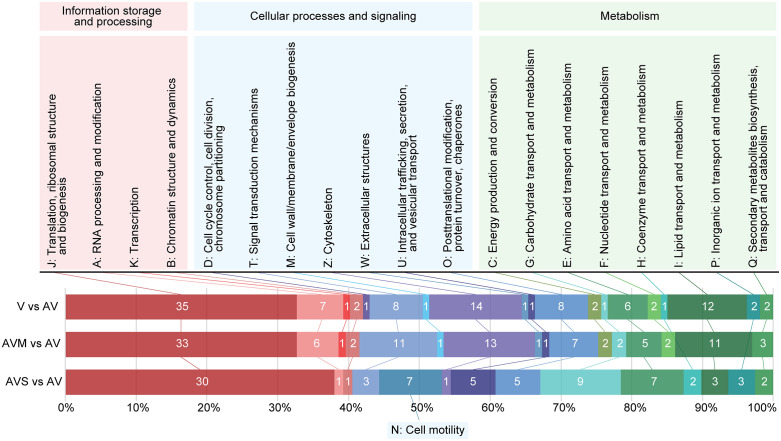
COG categories of differently expressed genes in studied cell lines. Proteins of differently expressed genes were categorized by eggNOG-mapper and their relative abundance is shown as horizontal stacked bar plots. Inside each bar, a total number of differentially expressed genes of that category is given. Note that category S: Function unknown and proteins without assigned COG were omitted for visualization purposes. For details see S2 Table.

To globally characterize the outcomes of these three differential gene expression analyses, we generated STRING networks (Figs 1 and S1 for the full network) and categorized genes into clusters of orthologous genes (COG) (Fig 2). While the COG categories revealed differences regardless of directionality of a change, the STRING diagrams showed directionally conserved changes in mRNA abundance between the different comparison sets and relationships between the transcripts involved.

Over 40% of differentially expressed genes in all three comparisons code for elements of information storage and processing with a larger percentage of these genes being linked to ribosomes and translation as shown in Figs 1 and 2 (COG category J with n = 383 genes; V vs AV: 35 DEGs, FDR = 1.40 × 10 ⁻ ⁷; AVM vs AV: 33 DEGs, FDR = 4.45 × 10 ⁻ ⁷; AVS vs AV: 30 DEGs, FDR = 1.07 × 10 ⁻ ⁹). A completely interconnected cluster of mRNAs all downregulated roughly two-fold in V, AVM, and AVS lines relative to AV was identified (Fig 1A). The tight spatial configuration and extensive associations of these transcripts is due to their identical ontology – they all encode ribosomal proteins. Furthermore, transcripts encoding additional ribosomal proteins, proteins needed for ribosome assembly, and proteins related to translation are among the shared genes exhibiting the same pattern of expression (Fig 1B-1D). Similarly to the universally regulated ribosomal proteins (Fig 1A), these transcripts are also downregulated approximately two-fold in the virulent lines, except for the eEF1A that was downregulated about six-fold in the AVS line. The latter finding is likely linked to the loss of *eEF1A* copies described in the previous section.

Exponential growth in culture that is unrestricted by nutritional constraints or other challenges of a host environment (for example, host defense mechanisms) naturally increases in rate. It is logical to assume that transition of cellular resources to support enhanced growth would include gene expression adaptations to increase the overall output of the protein translation machinery [78,79]. Conversely, unicellular organisms typically reduce translation in more restrictive circumstances (for example, upon reaching stationary phase in culture or in hosts, where conditions or nutritional resources may be less than ideal) [80,81]. In line with this, the most apparent gene expression feature of *L. major* cell lines associated with growth in a host versus in culture is a reduction in ribosomal proteins and proteins involved in ribosome functioning.

Changes to transcript abundances appearing after sand fly passage were not as extensive as those appearing after mouse passage, yet they did involve several different biological processes and pathways. In particular, the COG categories of cell motility (category N; 7 of only 13 annotated genes; FDR = 2.37 × 10 ⁻ ⁸) and intracellular trafficking (category U; present but not statistically enriched within AVS relative to AV differentially expressed genes) appeared completely or nearly confined to the AVS versus AV comparison, respectively (Fig 2). Cell motility upregulated transcripts in the AVS line (Fig 2) encoded proteins involved in the formation or function of the flagella. Also, the AVS versus AV changes to metabolic gene expression were centered around individual transcripts relating to carbohydrate transport and metabolism (category G; present but not statistically enriched). The most upregulated individual transcripts in AVS relative to the AV line were those related to synthesis of various phosphoglycans, or GPI-anchored proteins and other surface proteins, including transmembrane transporters (Fig 1C and S2C Table). While other comparisons also exhibited changes in transcripts related to the cell surface proteins, the identities of the individual transcripts involved did not overlap. Finally, a few transcripts involved in glycine biosynthesis were less abundant, while some facilitating its catabolism were more abundant. Additionally, some transcripts for stress response pathway proteins were mildly reduced in abundance, as were transcripts of a family of *L. major* specific nucleases (Fig 1C and S2C Table).

In contrast to changes resulting from passaging in sand flies, passaging in mice restored transcript abundances remarkably close to those of the original V line. Of all genes found to be upregulated in the V line relative to the AV line, 94% were also upregulated in the AVM line, typically to the same level (S2A and S2B Table). Of the transcripts found upregulated in only one of these comparisons, only two were upregulated more than 2.5-fold. Therefore, the pattern of transcript abundances in V and AVM lines was nearly identical. Compared to changes resulting from sand fly passaging, transcript abundance changes documented in the AVM (or V) line over AV were larger in magnitude. A few transcripts showed increases of eight- or even ten-fold (S2B Table). Finally, more transcripts were upregulated after passaging in mice (106, including hypothetical proteins and counting each copy of multicopy genes separately), relative to the number of transcripts upregulated following sand fly passaging (37) (S2 Table).

Topping the list of transcripts upregulated in V or AVM lines were surface proteins. One of them (*LmjF.04.0210* encoding a putative GPI-anchored protein) was upregulated 10-fold over the AV line. Altogether, transcripts of 11 proteins likely found on the parasite surface or equipped with a GPI anchor [82,83] were upregulated in the AVM line (S2B Table). They appear confined to the genus *Leishmania* or their nearest phylogenetic neighbors of the subfamily Leishmaniinae [84]. Many of these proteins possess leucine-rich repeat motifs. Their upregulation is expected, as they are implicated in macrophage infection. For example, the upregulated transcripts for proteophosphoglycans encode proteins PPG1–4 that play a role in macrophage binding preceding cell entry and amastigote replication [85,86]. Similarly, transcripts of eight proteins putatively involved in lipid metabolism or transport, including those encoding an acetylase, fatty acid elongase, and two ABC transporters, were upregulated (S2B Table). This is also expected, as the mechanisms to generate and move more surface proteins into place would also have to be more robust under these conditions. Cytoskeletal genes (Fig 2; category Z) were additionally enriched in both the V vs AV and AVM vs AV comparisons (14 and 13 differentially expressed genes of 149 total; FDR = 2.99 × 10 ⁻ ³ and 6.10 × 10 ⁻ ³, respectively), hinting at a host-specific component to cytoskeletal remodeling. Finally, despite GP63 protein levels and activity being downregulated in the AV line relative to the V, AVS, and AVM lines [16], we see only *gp63–4* was modestly upregulated in the AVM line (S1 Fig and S2B Table). Likewise, transcripts encoding LPG proteins that have been previously implicated in *Leishmania* virulence [17,87–89] were consistently expressed in all lines. At least for GP63, this is consistent with a previous hypothesis that expression and activity of GP63 may be influenced by regulatory mechanisms downstream of mRNA abundance, involving the coordinated interactions of the suite of surface proteins [23,90,91].

Specific regulation of the *L. major* proteins related to virulence or extended survival in different hosts is, at least partially, affected through modulation of transcript abundance. The logical question then becomes “how can transcripts be stabilized or destabilized under different environmental pressures”? One possibility is that transcripts might be differentially stabilized *via* ribonucleoprotein complexes (RNPs) [76]. The factors often driving RNP associations are RNA-binding proteins (RBPs) that typically bind 3′ UTRs of the mRNAs they regulate [92]. The related proteins LmjF.18.0170 and LmjF.18.0190 identified here are putative RBPs as they possess RNA-binding domains. The encoding genes are similar size and share 57% nucleotide identity. Their translated products retain a region that is 59% identical and 70% similar with two adjacent RNA recognition motifs identified by RRMScorer. The potential RNA motif for favorable binding was predicted to be U(A/U)UU. The transcript abundance of LmjF.18.0170 is modestly upregulated in both V and AVM lines and LmjF.18.0190 is four-fold upregulated in AVM (S2B Table). We hypothesized that these or other RBPs could be master regulators of the observed expression changes. An initial perusal of the first 100 nt of the 3′ UTRs of differentially expressed genes in the AVM line did not reveal enrichment of the U(A/U)UU motif. Furthermore, no other sequence motifs were determined to be enriched on more than a small (under 10%) subset of the differentially expressed genes in AVM and V lines. Therefore, the mechanism of transcript abundance regulation that is responsible for these changes remains an open question.

### Relative mitochondrial minicircle class abundances correlate with degree of virulence

In addition to changes to the nuclear genome, changes to the mitochondrial (kinetoplast) genome resulting from culture growth can impact *Leishmania* spp. fitness [93,94]. In this work, we found that the kinetoplast DNA region containing the protein-coding and rRNA-coding loci (the maxicircle CR) copy number and sequence is essentially identical in the *L. major* lines under study. However, the abundance of mature, translatable mitochondrial transcripts could play a role in a successful survival in *L. major* hosts. While expression changes to some mitochondrial mRNAs can be directly measured, others require specialized analysis. This is because most mitochondrial primary transcripts undergo massive RNA editing with uridine insertions and deletions to generate their translatable open reading frames [77,95–97]. This type of RNA editing reaction results in far more partially- than fully-edited, translatable products [98–100].

Here, we compared the overall transcription levels of all maxicircle-encoded genes and detailed editing maps of transcripts requiring editing. No significant differences between the four LV561-derived parasite lines were observed at the general transcription level (S2 Fig) or in the RNA editing of specific sites, with one exception. The surprising exception is the *G4* transcript (S3 Fig), the product of which has not yet been definitively attributed a function [101]. We observed *G4* to have a low overall transcription level and a lack of editing in the AV line, thus, the protein product of *G4* is unlikely to be generated in these parasites. In contrast, *G4* was more abundant and intensively edited in the V and AVM lines, while it was slightly less intensively edited in AVS cell line (S3 Fig). In contrast, extent of editing and the editing patterns of *RPS12* and *ND3* mRNAs were consistent across the lines. The changes in *G4* editing were the only observed cell line-specific differences to kinetoplast mRNA abundances, editing levels, or specific editing events that could impact *L. major* mitochondrial function in hosts.

**Fig 3:**
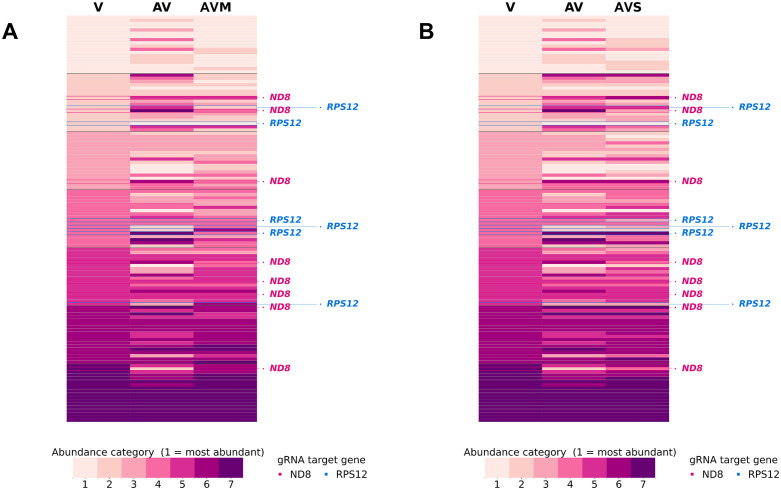
Minicircle sequence classes abundance by category. Heatmaps display copy numbers of minicircle classes measured by PRKM values divided into 7 abundance categories of equal size. Minicircle classes are sorted by decreasing abundance in the V sample. When relevant, we indicate with labels or triangular markers, the mRNA with homology to the gRNA encoded by this minicircle class. Blue, *RPS12* gRNA encoded; red, *ND8* gRNA encoded. **(A)** Comparison of V-AV-AVM lines; (**B**) comparison of V-AV-AVS lines.

In addition to the major protein machinery necessary to execute trypanosomatid mitochondrial RNA editing [95], hundreds of guide (g)RNAs are also needed to provide the specificity of the placement of insertions and deletions [102,103]. These gRNAs are encoded on many small circular mitochondrial DNA molecules that are independent of the larger molecules encoding the traditional elements of the mitochondrial genome. These “minicircles” are multicopy and organized into classes of near-identical molecules [45,104–106]. A potential regulator of gene-specific editing and overall mitochondrial genome informational content are the relative abundances of molecules of each class. We analyzed whether there were differences in the abundances of minicircle classes. Indeed, relative copy numbers of minicircles among classes were changed in the AV line compared to the numbers in the original V line (Fig 3). Notably, passage in mice and sand flies resulted in a close restoration of the relative abundances of the AVM and AVS line minicircles to what was observed in the V line, with some cell line-specific exceptions.

Notably, the observed lack of differences in editing patterns of cryptogenes make it unlikely that, in this case, minicircle class copy number mechanistically impacts editing in a direct or obvious manner. Nevertheless, we analyzed whether minicircle copy number abundance profiles were co-regulated based on the gRNA they encoded. This analysis requires computational reconstruction of the fully-edited open reading frame sequences of cryptogenes, to identify the edited mRNA corresponding to each minicircle’s encoded gRNAs. RNA-seq files were generated from total mRNA rather than mitochondrial-enriched mRNA. Therefore, mitochondrial read fractions were too low for reconstruction of a complete open reading frame for *G4*, the only cryptogene with evidence of differential editing among the LV561-derived lines. Reconstructions were possible for *ND8* and *RPS12*, however. We identified 9 and 6 minicircle-encoded gRNAs involved in their editing. As was the case with the total minicircle population, these minicircle class subsets also showed a general trend of minicircle abundance reversion when comparing V, AV, and host-passaged lines. Specifically, 7 out of 15 tracked minicircles returned to their original abundance category in the V–AV–AVM/AVS comparison, while 12 of 15 showed a clear tendency toward reversion (e.g., “high–low–high” or “low–high–low” patterns).

Despite trends for copy numbers of minicircle classes of *ND8* and *RPS12* editing cascades to reflect parasites’ virulence status, it is not at all clear that the alterations either promote or restrict editing. Not only were *ND8* and *RPS12* editing patterns and levels among the *L. major* lines themselves indistinguishable, but minicircles contributing gRNAs to these editing cascades did not exhibit a consistent directionality of change when compared among the parasite lines. Together, these observations suggest that minicircle abundance is not directly linked to mitochondrial gene expression or RNA editing efficiency. It is, thus, difficult to know what the biological outcome of these changes would be, or what might mechanistically drive minicircle class abundances. The mechanism for host passage-dependent maintenance or perturbation of minicircle class copy number warrants further investigation.

## Discussion

The genomic analyses presented here reveal factors potentially associated with virulence and/or persistence of *Leishmania* in the sand fly and mammalian hosts. While links between virulence and observed changes to mitochondrial gene expression and RNA editing remain opaque, the findings of nuclear genome CNV and gene expression patterns are logically consistent what is already known regarding virulence. The copy number analysis was notable particularly for the 12 kb segment of chromosome 35 overrepresented in the AV line that reverted to that of the original V line level upon subsequent host passages. This form of adaptive plasticity is the primary way gene dosage is regulated in *Leishmania* [64]. It has been commonly observed in *Leishmania* isolated from specific environments [107], likely facilitated by the abundance of repetitive sequence elements in their genomes [108]. CNV-driven increases of *Leishmania donovani* mRNAs associated with increased cell proliferation have been previously observed during transition to culture growth [64]. What our analysis in *L. major* adds is the fact that CNV of the impacted genes runs bidirectionally – copy number increases can be reversed when culture-adapted parasites repeatedly re-encounter the host environment. However, confirming the chromosome 35 region as a specific driver of increased protein translation and replication rate will require reverse genetic studies beyond the scope of this work. For instance, experimental restoration of the AV line chromosome 35 region copy number to that of the V line, possible through gene editing, would be expected to result in lower overall rates of translation and parasite replication rates.

Changes in mRNA abundance that are associated with *L. major* growth in culture or survival in a host appear to be important mechanisms governing these enhanced capabilities. Our results reveal that a likely major factor necessary for persistent survival in a host is somewhat pedestrian: a capacity for translation below the threshold of the maximal replication rate attainable in a nutrient-rich culture. This finding mirrors what is more broadly observed among intracellular pathogens such as *Trypanosoma cruzi* [109] and *Mycobacterium tuberculosis* [110] that, in some stages of infection, confine their own growth in order to limit their use of mammalian cell resources, avoid host cell death, and persist long-term. *Leishmania* is also known to have a similar “stringent response” [111,112]. Similarly, slower growth resulting from repressed translation in pathogens, both intracellular and extracellular, may support their persistence in mammalian hosts by keeping levels of non-host proteins below that which would trigger the innate immune response or nutritional immunity [113]. However, in the case of these LV561-derived *L. major* lines, the lower translation capacity phenotype cannot be mainly an immune evasion mechanism, as it appears near-equally when *Leishmania* replicates extracellularly in the gut of its sand fly host. Thus, a simple lack of nutrient resources is the force likely responsible for much of the difference in translation machinery abundance between parasites propagated in hosts *versus* culture, as it can explain the lower translational capacity in both hosts.

Beyond RNA abundance changes relating to translation, we note that gene expression alterations resulting from passaging in sand fly are rather modest and differ from patterns documented in both the original virulent and the mouse-passaged lines. This is not surprising, as the replicative life stage in sand fly and culture are the same (promastigote). However, nutrient availability in the sand fly gut is more erratic than in culture [114,115]. Another difference is the high proteolytic activity within the sand fly midgut, against which promastigotes express phosphoglycans [19,20,116,117]. Notably, transcripts of these types of proteins are increased in AVS, possibly because they play a protective role in the unique environment of the sand fly gut.

In contrast to the AVS vs AV comparison, transcript abundance differences between the V vs AV and AVM vs AV are more robust and nearly identical with each other. The robustness of the change and number of genes involved is likely due to the fact that the replicating parasites in the mammalian host are amastigotes in very different conditions than promastigotes in culture [118,119]. A lucrative lead in factors critical for mammalian host survival is the strong upregulation of transcripts of the *Leishmania*-specific ATG8-like gene arrays: *ATG8A* (3 genes), *ATG8B* (8 genes), and *ATG8C* (13 genes). Interestingly, this occurs despite constitutive expression of the *Leishmania* ortholog of the autophagosome-forming phosphatidylethanolamine-conjugated ATG8 that is more broadly conserved [120]. *Leishmania major* ATG8A and ATG8 are co-localized to autophagosome-like puncta in promastigotes, whereas ATG8B and ATG8C exhibit distinctly different localization. The latter proteins are diffusely present in the cytosol with a single concentrated punctum near the flagellar pocket and are unlikely to play a role in autophagy. Rather, upregulation of their transcripts upon passage in mice may indicate an increased need for the endocytic trafficking or recycling.

Although we have identified the RNAs that differ in abundance between AV and V or AVM lines, the upstream mechanisms responsible for these changes remain unknown. We did, however, discovered two upregulated RBPs that may contribute to some of the observed alterations in RNA abundance in the analyzed *L. major* lines. Additionally, the upregulation of at least five proteins with putative roles in signal transduction (two casein kinase I-like proteins, phosphoinositide phosphatase, NEK family Serine/threonine-protein kinase, and a calpain-like cysteine peptidase) in the AVM vs AV comparison suggests that protein stability or translational efficiency may also play a role in parasite survival.

Recent studies in *Leishmania* demonstrate that global translational reprogramming can occur during development and in response to stress and may include, for example, selective translation of subsets of transcripts mediated by RNA-binding proteins, differential ribosome loading, altered protein stability, and selection of subpopulations with enhanced translational capacity during mice passage. At the post-translational level, GP63 undergoes proteolytic processing, glycosylation, and membrane anchoring, which affect its maturation, stability, and activity, and its abundance is known to differ between life cycle stages, while LPG exhibits dynamic remodeling of surface expression during differentiation and contributes to immune evasion [121,122]. Collectively, findings related to GP63 and LPG expression [123,124] illustrate that mRNA analysis is incomplete as a strategy to identify *Leishmania* virulence factors. Ideally, for sufficiently abundant factors, quantitative proteomic analysis of these same parasite lines would be performed to both validate regulation suggested at the mRNA level and uncover virulence expression patterns regulated at levels beyond mRNA stability.

Arguably, the most perplexing finding of this study is the link between passage through a natural host and changes in the kinetoplast genome. This is because we are unable to demonstrate how or even whether the observed changes at the DNA or RNA level result in changes in abundance or sequence of mitochondrial-encoded proteins. Thus, we cannot yet identify the benefit of *G4* editing, or the maintenance of copy numbers of various minicircle classes in a specific order of relative abundance, on survival in either host. If greater sequencing depth would have revealed a full *G4* open reading frame consistent with that of its kinetoplastid homologues in V and AVM lines, disappearance of *G4* editing in the AV line may indicate that G4 is non-essential for growth in culture. This gene has been suggested to encode NADH dehydrogenase subunit 6 [101]; but in that case, it is unclear why other NADH dehydrogenase subunits are not similarly affected. Conversely, is there a role for a partially-edited *G4* mRNA unrelated to its translation into protein?

The restoration of minicircle copy number is even more puzzling. Our results show that except for *G4*, editing of mitochondrial cryptogene products is not altered among the strains. Thus, the impacts of differences in minicircle abundances on mitochondrial function are unlikely to be transmitted through differential RNA editing to alter abundances of cryptogene-encoded electron transport subunits. Despite this, do minicircle abundance ratios, through some unknown mechanism, still somehow tailor some mitochondrial function to the demands of life in a host? If so, some feature of the host environment must be the selective pressure that results in these specific abundance ratios between minicircle classes that is relaxed after long culture growth. Understanding that a variety of metabolic and oxidative processes are mitochondrial, three selective pressures come to mind. The first, as previously discussed, is restricted nutrient availability and less variety of nutrient types, which would occur in both sand fly and mouse macrophage hosts. Similarly, as the mitochondrial electron transport system utilized oxygen, lower oxygen availability (compared to cells in culture) may also impact this system [125]. Finally, *Leishmania* commonly encounters oxidative stress predominantly in host environments [126,127]. Regardless of the selective pressure, over time parasites with the most favourable minicircle class copy number ratios adapt to these stressors by expressing their noncoding RNAs in ratios that presumably influence one or more mitochondrial functions to completely unknown ways. This is an area ripe for future studies.

## Supporting information

S1 Table Genomic loci with copy number variation (CNV) across samples. Estimated integer loci copy number and their genomic positions are shown. CNV value of zero means deletion of the locus. If CNV regions contain protein coding genes, the gene names and their potential functional annotations (inferred from the TriTrypDB or BLASTP search of the *L. major* Friedlin reference genome) are listed in the column on the right. Tab LmjF.34.2380 documents bioinformatic analysis of *LmjF.34.2380* (gene presence or absence in genomes of Trypanosomatidae and TMP values in available RNA-seq datasets) and the protein it encodes (annotation, sequence, InterProScan, TMHMM, and DeepLoc predictions).
(XLSX)

S2 Table Differentially expressed genes. Lists of differentially expressed genes between AV and V, AVM, and AVS cell lines with FDR-adjusted *p*-value cut-off 0.001 and minimal absolute fold change greater than 2. Annotations are taken from the TriTrypDB *L. major* Friedlin genome annotation. Genes in bold were found to be differentially expressed in multiple sample comparisons. (A) V versus AV; (B) AVM versus AV; (C) AVS versus AV; (D) Statistical analysis by one-sided Fisher’s exact test (over-representation) with Benjamini-Hochberg FDR correction per comparison.
(XLSX)

S1 Fig Protein comparison of differently expressed genes in studied cell lines. The protein network as generated by STRING shows changes in mRNA abundance between the different cell lines. Nodes and edges represent *L. major* proteins and known or predicted interactions among them. Upward and downward arrows indicate up- or downregulation of the gene. Number of nodes: 266; number of edges: 653; PPI enrichment *p*-value 1 × 10^-16^.
(PDF)

S2 Fig Maxicircle coding region expression profiles. RNA-seq read coverage profiles of the maxicircle coding region of *L. major* LV561-derived cell lines. Three sequencing replicates are averaged and the coverage normalized to the expression of *ND9* gene (which has highest peak in each profile). Bottom track shows approximate homology-annotated gene boundaries, pan-edited and partially edited genes are shown in black and grey, respectively.
(PDF)

S3 Fig RNA editing of *G4*, *RPS12*, and *ND3* transcripts in studied lines. The scale in nucleotides is shown below the graphs. The number of inserted (+) or deleted (-) Us is demonstrated for every edited position.
(PDF)

## References

[1] Grimm F, Brun R, Jenni L. Promastigote infectivity in Leishmania infantum. Parasitol Res. 1991;77(3):185–91. doi: 10.1007/BF00930856 2047365

[2] Neal RA. Leishmania major: culture media, mouse strains, and promastigote virulence and infectivity. Exp Parasitol. 1984;57(3):269–73. doi: 10.1016/0014-4894(84)90100-0 6723897

[3] Dey T, Afrin F, Anam K, Ali N. Infectivity and virulence of Leishmania donovani promastigotes: a role for media, source, and strain of parasite. J Eukaryot Microbiol. 2002;49(4):270–4. doi: 10.1111/j.1550-7408.2002.tb00369.x 12188216

[4] Handman E, Hocking RE, Mitchell GF, Spithill TW. Isolation and characterization of infective and non-infective clones of Leishmania tropica. Mol Biochem Parasitol. 1983;7(2):111–26. doi: 10.1016/0166-6851(83)90039-7 6855810

[5] Kallinikova VD. Morphogenesis and virulence of Leishmania major in the process of long-term cultivation. Arch Protistenkd. 1992;141:327–34.

[6] Nasyrova RM, Kallinikova VD, Vafakulov SK, Nasyrov FS. The virulence and cytochemical properties of Leishmania major during long-term cultivation. Parazitologiia. 1993;27(3):233–41. 8321558

[7] Segovia M, Artero JM, Mellado E, Chance ML. Effects of long-term in vitro cultivation on the virulence of cloned lines of Leishmania major promastigotes. Ann Trop Med Parasitol. 1992;86(4):347–54. doi: 10.1080/00034983.1992.11812677 1463354

[8] Camara M, Navarro M, Segovia M. Evidence from genotypic and phenotypic markers that an attenuated line outgrows a virulent one in a mixed population of Leishmania major promastigotes cultured in vitro. Ann Trop Med Parasitol. 1995;89(5):477–84. doi: 10.1080/00034983.1995.11812980 7495361

[9] Katakura K, Kobayashi A. Enhancement of infectivity of Leishmania donovani promastigotes by serial mouse passages. J Parasitol. 1985;71(3):393–4. doi: 10.2307/3282033 4009354

[10] Sinha R, Raghwan CMM, Das S, Shadab M, et al. Genome Plasticity in Cultured Leishmania donovani: Comparison of Early and Late Passages. Front Microbiol. 2018;9:1279. doi: 10.3389/fmicb.2018.01279 30018594 PMC6037818

[11] Jha MK, Sarode AY, Bodhale N, Mukherjee D, Pandey SP, Srivastava N, et al. Development and Characterization of an Avirulent Leishmania major Strain. J Immunol. 2020;204(10):2734–53. doi: 10.4049/jimmunol.1901362 32245818

[12] Jacobson RL, Slutzky GM, Greenblatt CL, Schnur LF. Surface reaction of Leishmania. I. Lectin-mediated agglutination. Ann Trop Med Parasitol. 1982;76(1):45–52. 7082078

[13] Sádlová J, Svobodová M, Volf P. Leishmania major: effect of repeated passages through sandfly vectors or murine hosts. Ann Trop Med Parasitol. 1999;93(6):599–611. doi: 10.1080/00034989958104 10707105

[14] Kobets T, Čepičková M, Volkova V, Sohrabi Y, Havelková H, Svobodová M, et al. Novel Loci Controlling Parasite Load in Organs of Mice Infected With Leishmania major, Their Interactions and Sex Influence. Front Immunol. 2019;10:1083. doi: 10.3389/fimmu.2019.01083 31231359 PMC6566641

[15] Ciháková J, Volf P. Development of different Leishmania major strains in the vector sandflies Phlebotomus papatasi and P. duboscqi. Ann Trop Med Parasitol. 1997;91(3):267–79. doi: 10.1080/00034989761120 9229020

[16] Sádlová J, Volf P, Victoir K, Dujardin J-C, Votýpka J. Virulent and attenuated lines of Leishmania major: DNA karyotypes and differences in metalloproteinase GP63. Folia Parasitol. 2006;53(2):81–90. doi: 10.14411/fp.2006.011 16898121

[17] Späth GF, Epstein L, Leader B, Singer SM, Avila HA, Turco SJ, et al. Lipophosphoglycan is a virulence factor distinct from related glycoconjugates in the protozoan parasite Leishmania major. Proc Natl Acad Sci U S A. 2000;97(16):9258–63. doi: 10.1073/pnas.160257897 10908670 PMC16855

[18] Descoteaux A, Turco SJ. Glycoconjugates in Leishmania infectivity. Biochim Biophys Acta. 1999;1455(2–3):341–52. doi: 10.1016/s0925-4439(99)00065-4 10571023

[19] Sacks DL, Modi G, Rowton E, Späth G, Epstein L, Turco SJ, et al. The role of phosphoglycans in Leishmania-sand fly interactions. Proc Natl Acad Sci U S A. 2000;97(1):406–11. doi: 10.1073/pnas.97.1.406 10618431 PMC26676

[20] Svárovská A, Ant TH, Seblová V, Jecná L, Beverley SM, Volf P. Leishmania major glycosylation mutants require phosphoglycans (lpg2-) but not lipophosphoglycan (lpg1-) for survival in permissive sand fly vectors. PLoS Negl Trop Dis. 2010;4(1):e580. doi: 10.1371/journal.pntd.0000580 20084096 PMC2797086

[21] Kamhawi S Phlebotomine sand flies and *Leishmania* parasites: friends or foes? Trends Parasitol. 2006;22: 439–45. 16843727 10.1016/j.pt.2006.06.012

[22] Volf P, Myskova J. Sand flies and Leishmania: specific versus permissive vectors. Trends Parasitol. 2007;23(3):91–2. doi: 10.1016/j.pt.2006.12.010 17207663 PMC2839922

[23] Hajmová M, Chang K-P, Kolli B, Volf P. Down-regulation of gp63 in Leishmania amazonensis reduces its early development in Lutzomyia longipalpis. Microbes Infect. 2004;6(7):646–9. doi: 10.1016/j.micinf.2004.03.003 15158771

[24] Davies CR, Cooper AM, Peacock C, Lane RP, Blackwell JM. Expression of LPG and GP63 by different developmental stages of Leishmania major in the sandfly Phlebotomus papatasi. Parasitology. 1990;101 Pt 3:337–43. doi: 10.1017/s0031182000060522 2092290

[25] Dostálová A, Volf P. Leishmania development in sand flies: parasite-vector interactions overview. Parasit Vectors. 2012;5:276. doi: 10.1186/1756-3305-5-276 23206339 PMC3533922

[26] Sádlová J, Podešvová L, Bečvář T, Bianchi C, Gerasimov ES, Saura A, et al. Catalase impairs Leishmania mexicana development and virulence. Virulence. 2021;12(1):852–67. doi: 10.1080/21505594.2021.1896830 33724149 PMC7971327

[27] Yurchenko VY, Lukes J, Tesarová M, Jirků M, Maslov DA. Morphological discordance of the new trypanosomatid species phylogenetically associated with the genus crithidia. Protist. 2008;159(1):99–114. doi: 10.1016/j.protis.2007.07.003 17931968

[28] Zakharova A, Albanaz ATS, Opperdoes FR, Škodová-Sveráková I, Zagirova D, Saura A, et al. Leishmania guyanensis M4147 as a new LRV1-bearing model parasite: Phosphatidate phosphatase 2-like protein controls cell cycle progression and intracellular lipid content. PLoS Negl Trop Dis. 2022;16(6):e0010510. doi: 10.1371/journal.pntd.0010510 35749562 PMC9232130

[29] Andrews S. FastQC: a quality control tool for high throughput sequence data. 2023.

[30] Chen S. Ultrafast one-pass FASTQ data preprocessing, quality control, and deduplication using fastp. Imeta. 2023;2(2):e107. doi: 10.1002/imt2.107 38868435 PMC10989850

[31] Prjibelski A, Antipov D, Meleshko D, Lapidus A, Korobeynikov A. Using SPAdes de novo assembler. Curr Protoc Bioinformatics. 2020;70:e102. 10.1002/cpbi.102 32559359

[32] Tegenfeldt F, Kuznetsov D, Manni M, Berkeley M, Zdobnov EM, Kriventseva EV. OrthoDB and BUSCO update: annotation of orthologs with wider sampling of genomes. Nucleic Acids Res. 2025;53(D1):D516–22. doi: 10.1093/nar/gkae987 39535043 PMC11701741

[33] Li H, Durbin R. Fast and accurate short read alignment with Burrows-Wheeler transform. Bioinformatics. 2009;25(14):1754–60. doi: 10.1093/bioinformatics/btp324 19451168 PMC2705234

[34] Danecek P, Bonfield JK, Liddle J, Marshall J, Ohan V, Pollard MO, et al. Twelve years of SAMtools and BCFtools. Gigascience. 2021;10(2):giab008. doi: 10.1093/gigascience/giab008 33590861 PMC7931819

[35] Robinson JT, Thorvaldsdóttir H, Winckler W, Guttman M, Lander ES, Getz G, et al. Integrative genomics viewer. Nat Biotechnol. 2011;29(1):24–6. doi: 10.1038/nbt.1754 21221095 PMC3346182

[36] Garrison E, Marth GH. Haplotype-based variant detection from short-read sequencing. arXiv. 2012. 3907.

[37] Ginestet C. ggplot2: elegant graphics for data analysis. J R Stat Soc. 2011;174:245.

[38] Shanmugasundram A, Starns D, Böhme U, Amos B, Wilkinson PA, Harb OS, et al. TriTrypDB: An integrated functional genomics resource for kinetoplastida. PLoS Negl Trop Dis. 2023;17(1):e0011058. doi: 10.1371/journal.pntd.0011058 36656904 PMC9888696

[39] Quinlan AR. BEDTools: The Swiss-Army Tool for Genome Feature Analysis. Curr Protoc Bioinformatics. 2014;47:11.12.1-34. doi: 10.1002/0471250953.bi1112s47 25199790 PMC4213956

[40] Rausch T, Zichner T, Schlattl A, Stütz AM, Benes V, Korbel JO. DELLY: structural variant discovery by integrated paired-end and split-read analysis. Bioinformatics. 2012;28(18):i333–9. doi: 10.1093/bioinformatics/bts378 22962449 PMC3436805

[41] Auguie B, Antonov A (2017) gridExtra: miscellaneous functions for “grid” graphics.

[42] Klambauer G, Schwarzbauer K, Mayr A, Clevert D-A, Mitterecker A, Bodenhofer U, et al. cn.MOPS: mixture of Poissons for discovering copy number variations in next-generation sequencing data with a low false discovery rate. Nucleic Acids Res. 2012;40(9):e69. doi: 10.1093/nar/gks003 22302147 PMC3351174

[43] Camacho C, Coulouris G, Avagyan V, Ma N, Papadopoulos J, Bealer K, et al. BLAST+: architecture and applications. BMC Bioinformatics. 2009;10:421. doi: 10.1186/1471-2105-10-421 20003500 PMC2803857

[44] Gerasimov ES, Ramirez-Barrios R, Yurchenko V, Zimmer SL. Trypanosoma cruzi strain and starvation-driven mitochondrial RNA editing and transcriptome variability. RNA. 2022;28(7):993–1012. doi: 10.1261/rna.079088.121 35470233 PMC9202582

[45] Gerasimov ES, Gasparyan AA, Afonin DA, Zimmer SL, Kraeva N, Lukeš J, et al. Complete minicircle genome of Leptomonas pyrrhocoris reveals sources of its non-canonical mitochondrial RNA editing events. Nucleic Acids Res. 2021;49(6):3354–70. doi: 10.1093/nar/gkab114 33660779 PMC8034629

[46] Robinson MD, McCarthy DJ, Smyth GK. edgeR: a Bioconductor package for differential expression analysis of digital gene expression data. Bioinformatics. 2010;26(1):139–40. doi: 10.1093/bioinformatics/btp616 19910308 PMC2796818

[47] Ewels P, Magnusson M, Lundin S, Käller M. MultiQC: summarize analysis results for multiple tools and samples in a single report. Bioinformatics. 2016;32(19):3047–8. doi: 10.1093/bioinformatics/btw354 27312411 PMC5039924

[48] Dobin A, Gingeras TR. Optimizing RNA-Seq Mapping with STAR. Methods Mol Biol. 2016;1415:245–62. doi: 10.1007/978-1-4939-3572-7_13 27115637

[49] Okonechnikov K, Conesa A, García-Alcalde F. Qualimap 2: advanced multi-sample quality control for high-throughput sequencing data. Bioinformatics. 2016;32(2):292–4. doi: 10.1093/bioinformatics/btv566 26428292 PMC4708105

[50] Liao Y, Smyth GK, Shi W. featureCounts: an efficient general purpose program for assigning sequence reads to genomic features. Bioinformatics. 2014;30(7):923–30. doi: 10.1093/bioinformatics/btt656 24227677

[51] Love MI, Huber W, Anders S. Moderated estimation of fold change and dispersion for RNA-seq data with DESeq2. Genome Biol. 2014;15(12):550. doi: 10.1186/s13059-014-0550-8 25516281 PMC4302049

[52] Szklarczyk D, Kirsch R, Koutrouli M, Nastou K, Mehryary F, Hachilif R, et al. The STRING database in 2023: protein-protein association networks and functional enrichment analyses for any sequenced genome of interest. Nucleic Acids Res. 2023;51(D1):D638–46. doi: 10.1093/nar/gkac1000 36370105 PMC9825434

[53] Saura A, Zakharova A, Klocek D, Gerasimov ES, Butenko A, Macedo DH, et al. Elimination of LRVs Elicits Different Responses in Leishmania spp. mSphere. 2022;7(4):e0033522. doi: 10.1128/msphere.00335-22 35943162 PMC9429963

[54] Cantalapiedra CP, Hernández-Plaza A, Letunic I, Bork P, Huerta-Cepas J. eggNOG-mapper v2: Functional Annotation, Orthology Assignments, and Domain Prediction at the Metagenomic Scale. Mol Biol Evol. 2021;38(12):5825–9. doi: 10.1093/molbev/msab293 34597405 PMC8662613

[55] Roca-Martínez J, Dhondge H, Sattler M, Vranken WF. Deciphering the RRM-RNA recognition code: A computational analysis. PLoS Comput Biol. 2023;19(1):e1010859. doi: 10.1371/journal.pcbi.1010859 36689472 PMC9894542

[56] Madeira F, Madhusoodanan N, Lee J, Eusebi A, Niewielska A, Tivey ARN, et al. The EMBL-EBI Job Dispatcher sequence analysis tools framework in 2024. Nucleic Acids Res. 2024;52(W1):W521–5. doi: 10.1093/nar/gkae241 38597606 PMC11223882

[57] Bailey TL, Johnson J, Grant CE, Noble WS. The MEME Suite. Nucleic Acids Res. 2015;43(W1):W39-49. doi: 10.1093/nar/gkv416 25953851 PMC4489269

[58] Camacho E, González-de la Fuente S, Solana JC, Rastrojo A, Carrasco-Ramiro F, Requena JM, et al. Gene Annotation and Transcriptome Delineation on a De Novo Genome Assembly for the Reference Leishmania major Friedlin Strain. Genes (Basel). 2021;12(9):1359. doi: 10.3390/genes12091359 34573340 PMC8468144

[59] Ross MG, Russ C, Costello M, Hollinger A, Lennon NJ, Hegarty R, et al. Characterizing and measuring bias in sequence data. Genome Biol. 2013;14(5):R51. doi: 10.1186/gb-2013-14-5-r51 23718773 PMC4053816

[60] Treangen TJ, Salzberg SL. Repetitive DNA and next-generation sequencing: computational challenges and solutions. Nat Rev Genet. 2011;13(1):36–46. doi: 10.1038/nrg3117 22124482 PMC3324860

[61] DePristo MA, Banks E, Poplin R, Garimella KV, Maguire JR, Hartl C, et al. A framework for variation discovery and genotyping using next-generation DNA sequencing data. Nat Genet. 2011;43(5):491–8. doi: 10.1038/ng.806 21478889 PMC3083463

[62] Laffitte M-CN, Leprohon P, Papadopoulou B, Ouellette M. Plasticity of the Leishmania genome leading to gene copy number variations and drug resistance. F1000Res. 2016;5:2350. doi: 10.12688/f1000research.9218.1 27703673 PMC5031125

[63] Iantorno SA, Durrant C, Khan A, Sanders MJ, Beverley SM, Warren WC, et al. Gene Expression in Leishmania Is Regulated Predominantly by Gene Dosage. mBio. 2017;8(5):e01393-17. doi: 10.1128/mBio.01393-17 28900023 PMC5596349

[64] Bussotti G, Piel L, Pescher P, Domagalska MA, Rajan KS, Cohen-Chalamish S, et al. Genome instability drives epistatic adaptation in the human pathogen Leishmania. Proc Natl Acad Sci U S A. 2021;118(51):e2113744118. doi: 10.1073/pnas.2113744118 34903666 PMC8713814

[65] Rogers MB, Hilley JD, Dickens NJ, Wilkes J, Bates PA, Depledge DP, et al. Chromosome and gene copy number variation allow major structural change between species and strains of Leishmania. Genome Res. 2011;21(12):2129–42. doi: 10.1101/gr.122945.111 22038252 PMC3227102

[66] Alcoforado Diniz J, Chaves MM, Vaselek S, Miserani Magalhães RD, Ricci-Azevedo R, de Carvalho RVH, et al. Protein methyltransferase 7 deficiency in Leishmania major increases neutrophil associated pathology in murine model. PLoS Negl Trop Dis. 2021;15(3):e0009230. doi: 10.1371/journal.pntd.0009230 33651805 PMC7954300

[67] Ribeiro CV, Rocha BFB, Moreira D de S, Peruhype-Magalhães V, Murta SMF. Mannosyltransferase (GPI-14) overexpression protects promastigote and amastigote forms of Leishmania braziliensis against trivalent antimony. Parasit Vectors. 2019;12(1):60. doi: 10.1186/s13071-019-3305-2 30683152 PMC6346506

[68] da Silva Lira Filho A, Fajardo EF, Chang KP, Clément P, Olivier M. Leishmania Exosomes/Extracellular Vesicles Containing GP63 Are Essential for Enhance Cutaneous Leishmaniasis Development Upon Co-Inoculation of Leishmania amazonensis and Its Exosomes. Front Cell Infect Microbiol. 2022;11:709258. doi: 10.3389/fcimb.2021.709258 35186777 PMC8851419

[69] Lemley C, Yan S, Dole VS, Madhubala R, Cunningham ML, Beverley SM, et al. The Leishmania donovani LD1 locus gene ORFG encodes a biopterin transporter (BT1). Mol Biochem Parasitol. 1999;104(1):93–105. doi: 10.1016/s0166-6851(99)00132-2 10589984

[70] Dole VS, Myler PJ, Stuart KD, Madhubala R. Expression of biopterin transporter (BT1) protein in Leishmania. FEMS Microbiol Lett. 2002;208(1):89–91. doi: 10.1111/j.1574-6968.2002.tb11065.x 11934499

[71] Segovia M, Ortiz G. LD1 amplifications in Leishmania. Parasitol Today. 1997;13(9):342–8. doi: 10.1016/s0169-4758(97)01111-3 15275047

[72] Roy G, Kündig C, Olivier M, Papadopoulou B, Ouellette M. Adaptation of Leishmania cells to in vitro culture results in a more efficient reduction and transport of biopterin. Exp Parasitol. 2001;97(3):161–8. doi: 10.1006/expr.2001.4595 11312578

[73] de Toledo JS, Junqueira dos Santos AF, Rodrigues de Moura T, Antoniazi SA, Brodskyn C, Indiani de Oliveira C, et al. Leishmania (Viannia) braziliensis transfectants overexpressing the miniexon gene lose virulence in vivo. Parasitol Int. 2009;58(1):45–50. doi: 10.1016/j.parint.2008.09.006 18992366

[74] Navarro M, Maingon R, Hamers R, Segovia M. Dynamics and size polymorphisms of minichromosomes in Leishmania major LV-561 cloned lines. Mol Biochem Parasitol. 1992;55(1–2):65–74. doi: 10.1016/0166-6851(92)90127-6 1435877

[75] Sampaio MCR, Barbosa AF, Este MG, Pirmez C, Bello AR, Traub-Csekö YM. A 245kb mini-chromosome impacts on Leishmania braziliensis infection and survival. Biochem Biophys Res Commun. 2009;382(1):74–8. doi: 10.1016/j.bbrc.2009.02.128 19254695

[76] Clayton C. Regulation of gene expression in trypanosomatids: living with polycistronic transcription. Open Biol. 2019;9(6):190072. doi: 10.1098/rsob.190072 31164043 PMC6597758

[77] Maslov DA, Opperdoes FR, Kostygov AY, Hashimi H, Lukeš J, Yurchenko V. Recent advances in trypanosomatid research: genome organization, expression, metabolism, taxonomy and evolution. Parasitology. 2019;146(1):1–27. doi: 10.1017/S0031182018000951 29898792

[78] Wang T, Liang C, An Y, Xiao S, Xu H, Zheng M, et al. Engineering the Translational Machinery for Biotechnology Applications. Mol Biotechnol. 2020;62(4):219–27. doi: 10.1007/s12033-020-00246-y 32103426

[79] Firczuk H, Kannambath S, Pahle J, Claydon A, Beynon R, Duncan J, et al. An in vivo control map for the eukaryotic mRNA translation machinery. Mol Syst Biol. 2013;9:635. doi: 10.1038/msb.2012.73 23340841 PMC3564266

[80] Karpinets TV, Greenwood DJ, Sams CE, Ammons JT. RNA:protein ratio of the unicellular organism as a characteristic of phosphorous and nitrogen stoichiometry and of the cellular requirement of ribosomes for protein synthesis. BMC Biol. 2006;4:30. doi: 10.1186/1741-7007-4-30 16953894 PMC1574349

[81] Smith DR, Keeling PJ. Protists and the Wild, Wild West of Gene Expression: New Frontiers, Lawlessness, and Misfits. Annu Rev Microbiol. 2016;70:161–78. doi: 10.1146/annurev-micro-102215-095448 27359218

[82] Field MC, Menon AK, Cross GA. Developmental variation of glycosylphosphatidylinositol membrane anchors in Trypanosoma brucei. In vitro biosynthesis of intermediates in the construction of the GPI anchor of the major procyclic surface glycoprotein. J Biol Chem. 1992;267(8):5324–9. doi: 10.1016/s0021-9258(18)42769-x 1371998

[83] Denny PW, Field MC, Smith DF. GPI-anchored proteins and glycoconjugates segregate into lipid rafts in Kinetoplastida. FEBS Lett. 2001;491(1–2):148–53. doi: 10.1016/s0014-5793(01)02172-x 11226438

[84] Kostygov AY, Yurchenko V. Revised classification of the subfamily Leishmaniinae (Trypanosomatidae). Folia Parasitol. 2017;64:2017.020. doi: 10.14411/fp.2017.020 28783029

[85] Piani A, Ilg T, Elefanty AG, Curtis J, Handman E. Leishmania major proteophosphoglycan is expressed by amastigotes and has an immunomodulatory effect on macrophage function. Microbes Infect. 1999;1(8):589–99. doi: 10.1016/s1286-4579(99)80058-6 10611735

[86] Giraud E, Lestinova T, Derrick T, Martin O, Dillon RJ, Volf P, et al. Leishmania proteophosphoglycans regurgitated from infected sand flies accelerate dermal wound repair and exacerbate leishmaniasis via insulin-like growth factor 1-dependent signalling. PLoS Pathog. 2018;14(1):e1006794. doi: 10.1371/journal.ppat.1006794 29352310 PMC5792026

[87] Beverley SM, Turco SJ. Lipophosphoglycan (LPG) and the identification of virulence genes in the protozoan parasite Leishmania. Trends Microbiol. 1998;6(1):35–40. doi: 10.1016/S0966-842X(97)01180-3 9481823

[88] Späth GF, Garraway LA, Turco SJ, Beverley SM. The role(s) of lipophosphoglycan (LPG) in the establishment of Leishmania major infections in mammalian hosts. Proc Natl Acad Sci U S A. 2003;100(16):9536–41. doi: 10.1073/pnas.1530604100 12869694 PMC170953

[89] Späth GF, Lye L-F, Segawa H, Sacks DL, Turco SJ, Beverley SM. Persistence without pathology in phosphoglycan-deficient Leishmania major. Science. 2003;301(5637):1241–3. doi: 10.1126/science.1087499 12947201

[90] Olivier M, Atayde VD, Isnard A, Hassani K, Shio MT. Leishmania virulence factors: focus on the metalloprotease GP63. Microbes Infect. 2012;14(15):1377–89. doi: 10.1016/j.micinf.2012.05.014 22683718

[91] Yao C, Donelson JE, Wilson ME. The major surface protease (MSP or GP63) of Leishmania sp. Biosynthesis, regulation of expression, and function. Mol Biochem Parasitol. 2003;132(1):1–16. doi: 10.1016/s0166-6851(03)00211-1 14563532

[92] Street LA, Rothamel KL, Brannan KW, Jin W, Bokor BJ, Dong K, et al. Large-scale map of RNA-binding protein interactomes across the mRNA life cycle. Mol Cell. 2024;84(19):3790-3809.e8. doi: 10.1016/j.molcel.2024.08.030 39303721 PMC11530141

[93] Thiemann OH, Maslov DA, Simpson L. Disruption of RNA editing in Leishmania tarentolae by the loss of minicircle-encoded guide RNA genes. EMBO J. 1994;13(23):5689–700. doi: 10.1002/j.1460-2075.1994.tb06907.x 7988566 PMC395534

[94] Simpson L, Douglass SM, Lake JA, Pellegrini M, Li F. Comparison of the Mitochondrial Genomes and Steady State Transcriptomes of Two Strains of the Trypanosomatid Parasite, Leishmania tarentolae. PLoS Negl Trop Dis. 2015;9(7):e0003841. doi: 10.1371/journal.pntd.0003841 26204118 PMC4512693

[95] Aphasizheva I, Alfonzo J, Carnes J, Cestari I, Cruz-Reyes J, Göringer HU, et al. Lexis and Grammar of Mitochondrial RNA Processing in Trypanosomes. Trends Parasitol. 2020;36(4):337–55. doi: 10.1016/j.pt.2020.01.006 32191849 PMC7083771

[96] Read LK, Lukeš J, Hashimi H. Trypanosome RNA editing: the complexity of getting U in and taking U out. Wiley Interdiscip Rev RNA. 2016;7(1):33–51. doi: 10.1002/wrna.1313 26522170 PMC4835692

[97] Aphasizhev R, Aphasizheva I. Mitochondrial RNA editing in trypanosomes: small RNAs in control. Biochimie. 2014;100:125–31. doi: 10.1016/j.biochi.2014.01.003 24440637 PMC4737708

[98] Simpson RM, Bruno AE, Bard JE, Buck MJ, Read LK. High-throughput sequencing of partially edited trypanosome mRNAs reveals barriers to editing progression and evidence for alternative editing. RNA. 2016;22(5):677–95. doi: 10.1261/rna.055160.115 26908922 PMC4836643

[99] Gerasimov ES, Afonin DA, Korzhavina OA, Lukeš J, Low R, Hall N, et al. Mitochondrial RNA editing in Trypanoplasma borreli: New tools, new revelations. Comput Struct Biotechnol J. 2022;20:6388–402. doi: 10.1016/j.csbj.2022.11.023 36420151 PMC9679448

[100] Carnes J, McDermott SM, Stuart K. RNA editing catalytic complexes edit multiple mRNA sites non-processively in Trypanosoma brucei. Mol Biochem Parasitol. 2023;256:111596. doi: 10.1016/j.molbiopara.2023.111596 37742784 PMC11913371

[101] Gerasimov ES, Afonin DA, Škodová-Sveráková I, Saura A, Trusina N, Gahura O, et al. Evolutionary divergent kinetoplast genome structure and RNA editing patterns in the trypanosomatid Vickermania. Proc Natl Acad Sci U S A. 2025;122(15):e2426887122. doi: 10.1073/pnas.2426887122 40203041 PMC12012515

[102] Liu S, Wang H, Li X, Zhang F, Lee JKJ, Li Z, et al. Structural basis of gRNA stabilization and mRNA recognition in trypanosomal RNA editing. Science. 2023;381(6653):eadg4725. doi: 10.1126/science.adg4725 37410820 PMC10704856

[103] Kable ML, Seiwert SD, Heidmann S, Stuart K. RNA editing: a mechanism for gRNA-specified uridylate insertion into precursor mRNA. Science. 1996;273(5279):1189–95. doi: 10.1126/science.273.5279.1189 8703045

[104] Iurchenko VI, Kolesnikov AA. Minicircular kinetoplast DNA from Trypanosomatidae. Mol Biol (Mosk). 2001;35(1):3–13. doi: 10.1023/a:1004813414607 11234380

[105] Cooper S, Wadsworth ES, Schnaufer A, Savill NJ. Organization of minicircle cassettes and guide RNA genes in Trypanosoma brucei. RNA. 2022;28(7):972–92. doi: 10.1261/rna.079022.121 35414587 PMC9202587

[106] Cooper S, Wadsworth ES, Ochsenreiter T, Ivens A, Savill NJ, Schnaufer A. Assembly and annotation of the mitochondrial minicircle genome of a differentiation-competent strain of Trypanosoma brucei. Nucleic Acids Res. 2019;47(21):11304–25. doi: 10.1093/nar/gkz928 31665448 PMC6868439

[107] Bussotti G, Gouzelou E, Côrtes Boité M, Kherachi I, Harrat Z, Eddaikra N, et al. Leishmania Genome Dynamics during Environmental Adaptation Reveal Strain-Specific Differences in Gene Copy Number Variation, Karyotype Instability, and Telomeric Amplification. mBio. 2018;9(6):e01399-18. doi: 10.1128/mBio.01399-18 30401775 PMC6222132

[108] Späth GF, Piel L, Pescher P. Leishmania genomic adaptation: more than just a 36-body problem. Trends Parasitol. 2025;41(6):441–8. doi: 10.1016/j.pt.2025.04.002 40316476

[109] Barrett MP, Kyle DE, Sibley LD, Radke JB, Tarleton RL. Protozoan persister-like cells and drug treatment failure. Nat Rev Microbiol. 2019;17(10):607–20. doi: 10.1038/s41579-019-0238-x 31444481 PMC7024564

[110] Prusa J, Zhu DX, Stallings CL. The stringent response and Mycobacterium tuberculosis pathogenesis. Pathog Dis. 2018;76(5):fty054. doi: 10.1093/femspd/fty054 29947752 PMC7191866

[111] Saunders EC, Ng WW, Kloehn J, Chambers JM, Ng M, McConville MJ. Induction of a stringent metabolic response in intracellular stages of Leishmania mexicana leads to increased dependence on mitochondrial metabolism. PLoS Pathog. 2014;10(1):e1003888. doi: 10.1371/journal.ppat.1003888 24465208 PMC3900632

[112] Kloehn J, Saunders EC, O’Callaghan S, Dagley MJ, McConville MJ. Characterization of metabolically quiescent Leishmania parasites in murine lesions using heavy water labeling. PLoS Pathog. 2015;11(2):e1004683. doi: 10.1371/journal.ppat.1004683 25714830 PMC4340956

[113] Palmer LD, Skaar EP. Transition Metals and Virulence in Bacteria. Annu Rev Genet. 2016;50:67–91. doi: 10.1146/annurev-genet-120215-035146 27617971 PMC5125913

[114] Moraes CS, Lucena SA, Moreira BHS, Brazil RP, Gontijo NF, Genta FA. Relationship between digestive enzymes and food habit of Lutzomyia longipalpis (Diptera: Psychodidae) larvae: Characterization of carbohydrases and digestion of microorganisms. J Insect Physiol. 2012;58(8):1136–45. doi: 10.1016/j.jinsphys.2012.05.015 22684112

[115] Ferreira TN, Latgé SGC, Ramos TD, Demidoff FC, Cunha-Júnior EF, Costa PRR, et al. Evaluation of sugar meal administered anti-Leishmania compounds on the vectorial capacity of the vector, Lutzomyia longipalpis. PLoS One. 2025;20(6):e0325178. doi: 10.1371/journal.pone.0325178 40560960 PMC12194183

[116] Rogers ME. The role of leishmania proteophosphoglycans in sand fly transmission and infection of the Mammalian host. Front Microbiol. 2012;3:223. doi: 10.3389/fmicb.2012.00223 22754550 PMC3384971

[117] Ilg T, Handman E, Stierhof YD. Proteophosphoglycans from Leishmania promastigotes and amastigotes. Biochem Soc Trans. 1999;27(4):518–25. doi: 10.1042/bst0270518 10917633

[118] Silva-Moreira AL, Serravite AM, Rios-Barros LV, de Menezes JPB, Horta MF, Castro-Gomes T. New insights into the life cycle, host cell tropism, and infection amplification of Leishmania spp. Infect Immun. 2025;93(7):e0012325. doi: 10.1128/iai.00123-25 40512038 PMC12234434

[119] Burchmore RJ, Barrett MP. Life in vacuoles--nutrient acquisition by Leishmania amastigotes. Int J Parasitol. 2001;31(12):1311–20. doi: 10.1016/s0020-7519(01)00259-4 11566299

[120] Williams RAM, Woods KL, Juliano L, Mottram JC, Coombs GH. Characterization of unusual families of ATG8-like proteins and ATG12 in the protozoan parasite Leishmania major. Autophagy. 2009;5(2):159–72. doi: 10.4161/auto.5.2.7328 19066473 PMC2642932

[121] Mandell MA, Beatty WL, Beverley SM. Quantitative single-cell analysis of Leishmania major amastigote differentiation demonstrates variably extended expression of the lipophosphoglycan (LPG) virulence factor in different host cell types. PLoS Negl Trop Dis. 2022;16(10):e0010893. doi: 10.1371/journal.pntd.0010893 36302046 PMC9642900

[122] Ghosh SK, Shukla D, Mahor H, Srivastava SK, Bodhale N, Banerjee R, et al. Leishmania surface molecule lipophosphoglycan-TLR2 interaction moderates TPL2-mediated TLR2 signalling for parasite survival. Immunology. 2024;171(1):117–30. doi: 10.1111/imm.13702 37849037

[123] da Silva LC, Aoki JI, Floeter-Winter LM. Finding correlations between mRNA and protein levels in Leishmania development: is there a discrepancy?. Front Cell Infect Microbiol. 2022;12:852902. 35903202 10.3389/fcimb.2022.852902 PMC9318571

[124] Gupta AK, Das S, Kamran M, Ejazi SA, Ali N. The pathogenicity and virulence of Leishmania - interplay of virulence factors with host defenses. Virulence. 2022;13(1):903–35. doi: 10.1080/21505594.2022.2074130 35531875 PMC9154802

[125] Mahnke A, Meier RJ, Schatz V, Hofmann J, Castiglione K, Schleicher U, et al. Hypoxia in Leishmania major skin lesions impairs the NO-dependent leishmanicidal activity of macrophages. J Invest Dermatol. 2014;134(9):2339–46. doi: 10.1038/jid.2014.121 24583949

[126] Bussotti G, Li B, Pescher P, Vojtkova B, Louradour I, Pruzinova K, et al. Leishmania allelic selection during experimental sand fly infection correlates with mutational signatures of oxidative DNA damage. Proc Natl Acad Sci U S A. 2023;120(10):e2220828120. doi: 10.1073/pnas.2220828120 36848551 PMC10013807

[127] Nunes AP, Dos Santos-Destro YM, Rodrigues ACJ, Detoni MB, Cruz EMS, Berbert GS, et al. Under pressure: Updated insights into the mechanisms of Leishmania’s defense in response to oxidative stress. Life Sci. 2025;377:123779. doi: 10.1016/j.lfs.2025.123779 40460927

