# Supplementary figures and images for: Leishmania major virulence attenuation in vitro: An old conundrum revisited in the omics era
Source: PLoS Negl Trop Dis. 2026 May 29;20(5):e0014387. doi: 10.1371/journal.pntd.0014387 (PMC13221023; doi:10.1371/journal.pntd.0014387)

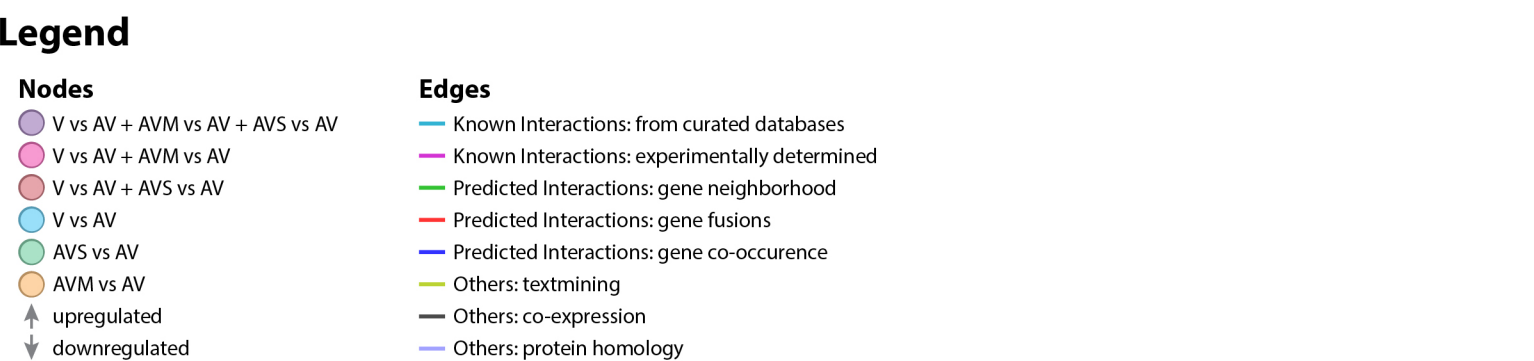

Supplement: S1 Fig — The protein network as generated by STRING shows changes in mRNA abundance between the different cell lines. Nodes and edges represent L. major proteins and known or predicted interactions among them. Upward and downward arrows indicate up- or downregulation of the gene. Number of nodes: 266; number of edges: 653; PPI enrichment p-value 1 × 10^-16^. (PDF) [file pntd.0014387.s003.pdf]

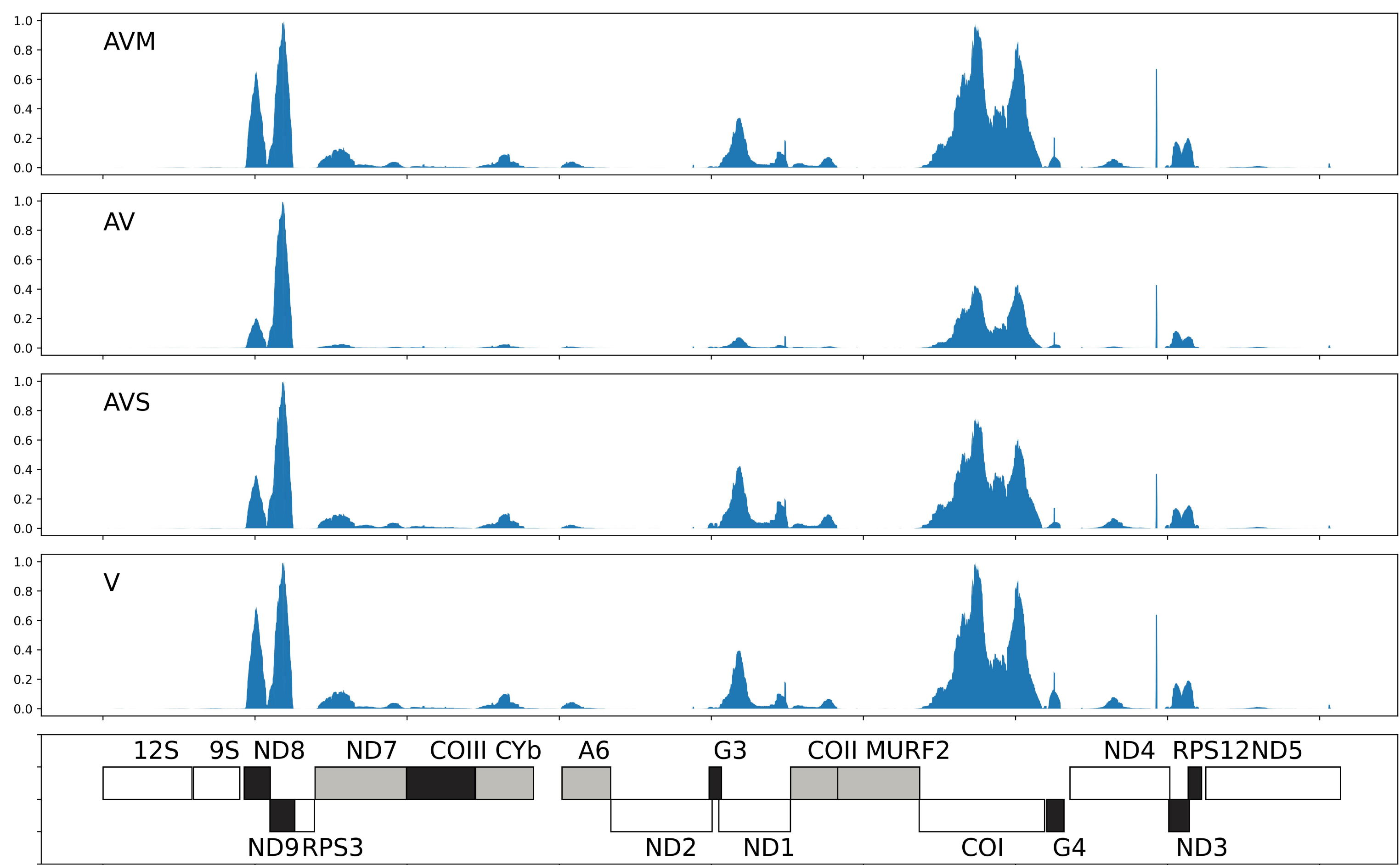

Supplement: S2 Fig — RNA-seq read coverage profiles of the maxicircle coding region of L. major LV561-derived cell lines. Three sequencing replicates are averaged and the coverage normalized to the expression of ND9 gene (which has highest peak in each profile). Bottom track shows approximate homology-annotated gene boundaries, pan-edited and partially edited genes are shown in black and grey, respectively. (PDF) [file pntd.0014387.s004.pdf]
