# Supplementary material for: Leishmania major virulence attenuation in vitro: An old conundrum revisited in the omics era
Source: PLoS Negl Trop Dis. 2026 May 29;20(5):e0014387. doi: 10.1371/journal.pntd.0014387 (PMC13221023; doi:10.1371/journal.pntd.0014387)

A

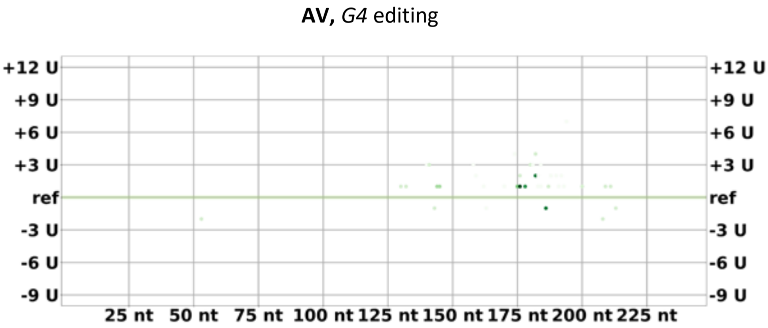
AV, G4 editing
+12 U
+9 U
+6 U
+3 U
ref
-3 U
-6 U
-9 U
25 nt 50 nt 75 nt 100 nt 125 nt 150 nt 175 nt 200 nt 225 nt

B

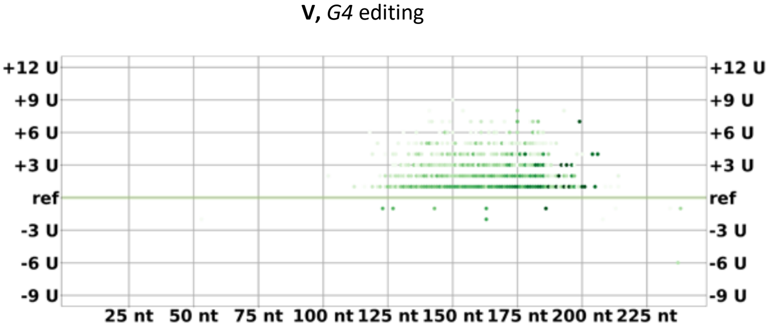
V, G4 editing
+12 U
+9 U
+6 U
+3 U
ref
-3 U
-6 U
-9 U
25 nt 50 nt 75 nt 100 nt 125 nt 150 nt 175 nt 200 nt 225 nt

C

AVM, *G4* editing

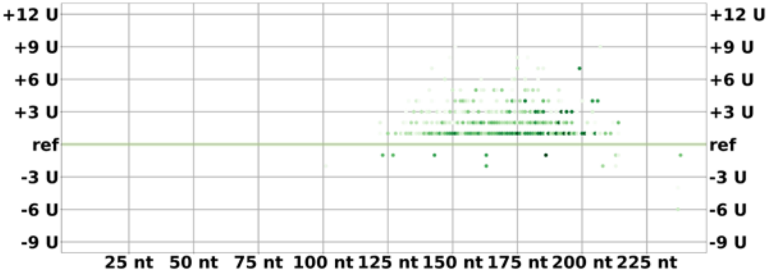

D

AVS, *G4* editing

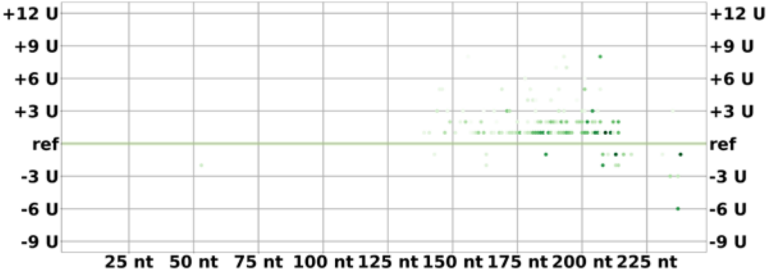

E

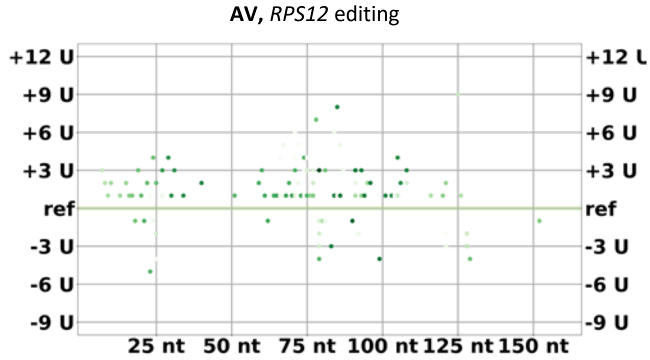
AV, *RPS12* editing
+12 U
+9 U
+6 U
+3 U
ref
-3 U
-6 U
-9 U
25 nt
50 nt
75 nt
100 nt
125 nt
150 nt

F

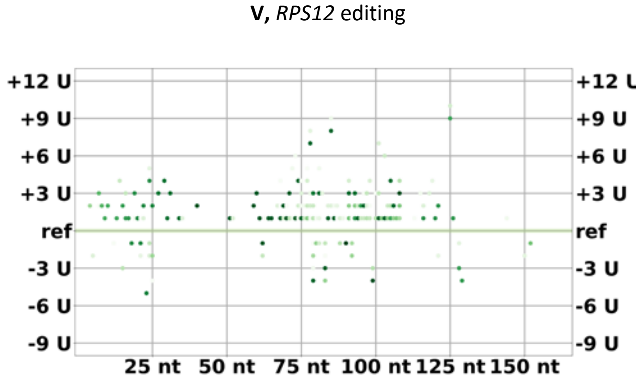
V, *RPS12* editing
+12 U
+9 U
+6 U
+3 U
ref
-3 U
-6 U
-9 U
25 nt
50 nt
75 nt
100 nt
125 nt
150 nt

G

AVM, ***RPS12*** **editing**

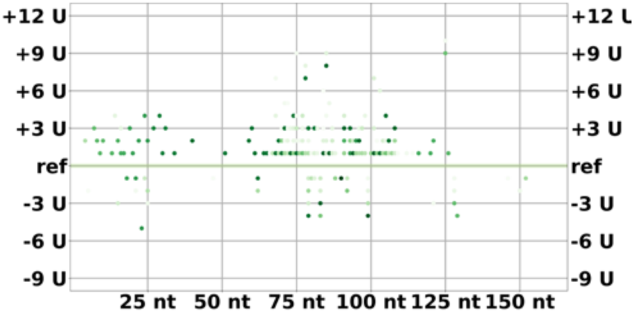

AVS, ***RPS12*** **editing**

H

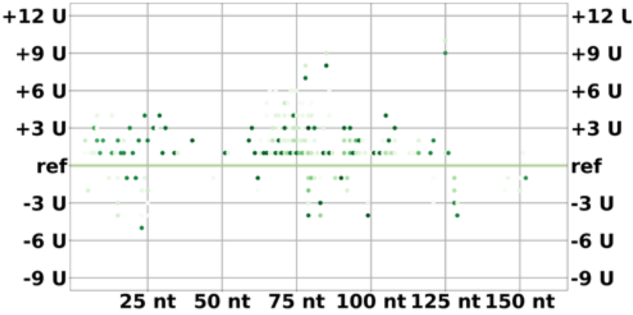

I

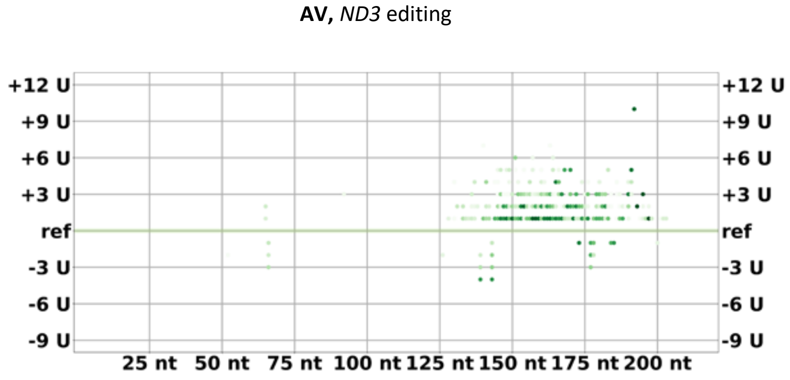
AV, ND3 editing
+12 U
+9 U
+6 U
+3 U
ref
-3 U
-6 U
-9 U
25 nt
50 nt
75 nt
100 nt
125 nt
150 nt
175 nt
200 nt

J

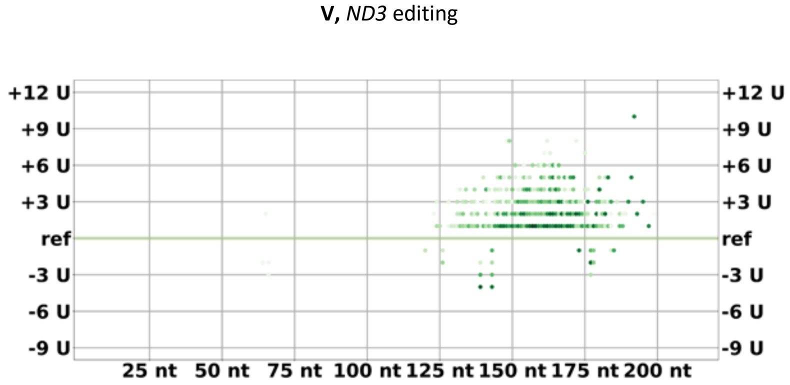
V, ND3 editing
+12 U
+9 U
+6 U
+3 U
ref
-3 U
-6 U
-9 U
25 nt
50 nt
75 nt
100 nt
125 nt
150 nt
175 nt
200 nt

K

AVM, ***ND3*** **editing**

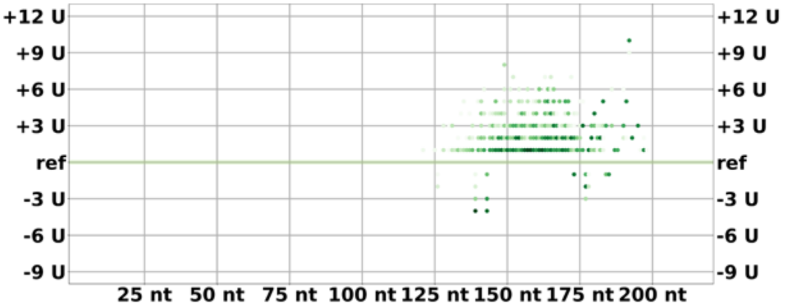

AVS, ***ND3*** **editing**

L

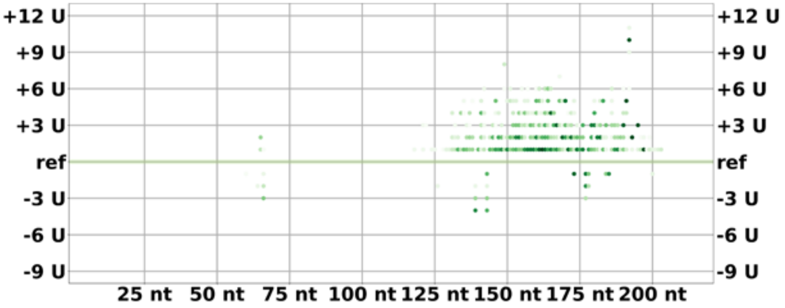

Supplement: S3 Fig — The scale in nucleotides is shown below the graphs. The number of inserted (+) or deleted (-) Us is demonstrated for every edited position. (PDF) [file pntd.0014387.s005.pdf]
